# Polarized super-resolution structural imaging inside amyloid fibrils using Thioflavine T

**DOI:** 10.1038/s41598-017-12864-9

**Published:** 2017-10-02

**Authors:** Haitham A. Shaban, Cesar A. Valades-Cruz, Julien Savatier, Sophie Brasselet

**Affiliations:** 1 Aix Marseille Univ, CNRS, Centrale Marseille, Institut Fresnel, F-13013, Marseille, France; 2 Spectroscopy Department, National Research Centre, El-Bohouth Str., 12622, Dokki, Giza Egypt; 3 Laboratoire de Biologie Moléculaire Eucaryote (LBME); Centre de Biologie Intégrative (CBI); Université de Toulouse; CNRS; UPS; 31062, Toulouse, France; 4 Present Address: Institut Curie - Centre de Recherche, PSL Research University, Endocytic Trafficking and Intracellular Delivery Laboratory, 75248, Paris cedex 05, France

## Abstract

Thioflavin T (ThT) is standardly used as a fluorescent marker to detect aggregation of amyloid fibrils by conventional fluorescence microscopy, including polarization resolved imaging that brings information on the orientational order of the fibrils. These techniques are however diffraction limited and cannot provide fine structural details at the fibrils scales of 10–100 nm, which lie beyond the diffraction limit. In this work, we evaluate the capacity of ThT to photoswitch when bound to insulin amyloids by adjusting the redox properties of its environment. We demonstrate that on-off duty cycles, intensity and photostability of the ThT fluorescence emission under adequate buffer conditions permit stochastic super-resolution imaging with a localization precision close to 20 nm. We show moreover that signal to noise conditions allow polarized orientational imaging of single ThT molecules, which reveals ultra-structure signatures related to protofilaments twisting within amyloid fibrils.

## Introduction

Amyloid aggregates, which originate from protein misfolding as a starting point for aggregation and plaque formations, are known to be at the origin of crucial processes responsible for neurodegenerative diseases. Thioflavin T (ThT), a small benzothiazole fluorescent compound, is commonly used for imaging amyloid fibrils by exploiting its capacity to generate high fluorescence emission when bound to amyloid in specific sites^1^. Fluorescence-based optical imaging techniques have used ThT to study the formation and growth processes of amyloid fibrils^2,3^, as well as for the diagnosis of plaque formation in diseases related to protein disorder^4,5^. Moreover, fluorescence anisotropy and fluorescence polarized microscopy have evidenced the high orientational order of ThT in amyloids and its ability to report indirectly the organization and structure of fibrils *in vitro* ^6–8^.

Although fluorescence optical imaging is a powerful tool to gain insight into the formation and the aggregation of amyloids, it is still limited to spatial scales of the order of the optical diffraction limit, e.g. about 200 nm. This resolution scale is however inappropriate for monitoring the supra-molecular architecture of the amyloid aggregates, which size is around 10–100 nm from electron and atomic force microscopies^9,10^. Individual filaments made of repeated β-sheets are known in particular to arrange into protofilaments that twist around each other in a helical ribbon, which pitch size varies depending on the number of filaments involved^8^. Accessing such scales in non-invasive and ideally *in-vivo* conditions is essential for understanding the molecular mechanisms responsible for the formation of amyloids and their functional consequences in neurodegenerative diseases.

Super resolution imaging based on single molecule localization has recently brought a determining step to overcome this limitation, using the stochastic nature of emission from isolated molecules localized with nanometric accuracy^11,12^. Direct Stochastic Optical Reconstruction Microscopy (dSTORM) uses in particular the capacity of fluorophores to photoswitch from dark to bright fluorescence states, at a rate accessible in imaging conditions^13^. This method has been used to study the high resolution morphology and aggregates formation of different types of amyloids such as amyloid beta (Aβ)^14,15^,α-synuclein^16–18^ and Huntingtin protein^19–21^, in both *in vivo* conditions and in fixed cells, with a resolution down to 20 nm. These approaches however have so far relied on either immunolabeling of the amyloid fibrils or the covalent binding of monomeric proteins by fluorescent organic dye. Immunolabeling reports only apparent fibrils organization due to the non-negligible size of the antibodies^14^. Fluorescent labeled monomers have overcome this limitation, reaching better than 20 nm resolution for studying the aggregation process of α-synuclein fibrils^16^. Covalent labeling however requires a tight control of labeling density to not interfere with the biological function leading to amyloid aggregates formation^16^. Some of those limitations have been overcome by binding-activated localization microscopy (BALM) by mixing α-synuclein amyloid fibrils with fluorescent target-binding probes in buffer solution at low concentration. Whenever the fluorophore binds to amyloid, the target structure becomes highly fluorescent and can be localized with few nanometer accuracy^22^. BALM however still suffers from nonspecific binding and spontaneous activation of probes at high labeling concentration. At last none of those super-resolution imaging approaches is appropriate for structural imaging of amyloids^6–8^. Indeed reporting filaments orientational organization using the polarization dependence of fluorescence signals requires a label that binds amyloids in a non-flexible way, keeping a high degree of fidelity between fluorophore orientation and local filaments organization.

In this work, we report the use of ThT for super-resolution imaging of amyloids, with the goal to benefit from its intrinsic intercalating nature that provides a high degree of order, potentially reporting the local ultra-structure of fibril without profound modification of its function. The study is performed on insulin amyloids, which is used as a common model for fibrillogenesis^9^. When bound to amyloids, ThT is stabilized by a close by β-sheet interface both sterically and electronically by aromatic interactions^23,24^, providing some degree of orientational constraint as measured by polarized microscopy^6–8^. In this bound form, ThT exhibits a conjugated nature similar to many organic dyes, and is thus a candidate for photo-switching since intersystem crossing pathways may occur, enhanced by proper buffer conditions. In what follows, we quantify these properties in buffer conditions usually employed for dSTORM, and show the potential of ThT as a photo-switchable probe for dSTORM. We exploit moreover the possibilities of polarized dSTORM (P-dSTORM) to quantify the oriented nature of ThT within the amyloid binding sites, analyzing their fluorescence polarization projected onto two orthogonal analyzer directions^25^. Polarization splitting presents some advantages with respect to other existing techniques^26,27^ in relatively low signal to noise conditions. Using this method we observe non-negligible angular fluctuations undergone by single ThT molecules within the measurement integration time, providing an insight into disorder mechanisms^28–30^ that hide behind ensemble polarized fluorescence experiments previously reported^7,8^. Correcting polarized data from such fluctuations, we provide not only morphological imaging, but also structural information using ThT as a reporter of the sub-fibrils directions that constitute amyloid aggregates.

## Results and Discussion

Super-resolution dSTORM imaging relies on the measurement of localization of isolated molecules with high precision, followed by image reconstruction based on a collection of a high number of detection events^13^. Two essential properties of photo-switchable dyes are fundamental for achieving high quality in dSTORM imaging^31^: (i) a high number of photons detected per switching event, which ensures a high localization precision^32,33^, and (ii) a low duty cycle (fraction of time a fluorophore spends in the on state ${{\rm{\tau }}}_{on}$ with respect to the off state ${{\rm{\tau }}}_{off}$: ${{\rm{\tau }}}_{on}/{{\rm{\tau }}}_{off}$), which ensures a high probability of localization^34,35^. A third relevant property is photostability, which ensures a high number of detectable molecules over a given measurement time^36^. Altogether, a dye which exhibits a high intensity per switching event, a low duty cycle, and a high photostability is adequate for constructing high quality dSTORM images^31^.

These properties have been optimized for conjugated dSTORM dyes thanks to the use of appropriate dosing of redox chemical reactions and oxygen removal^13,37^. In this work we test the photo-switching properties of ThT by applying two different reducing agents, mercaptoethylamine (MEA) and ascorbic acid (AA), in presence of an oxygen scavenging system based on glucose oxidase/catalase.

**Figure 1:**
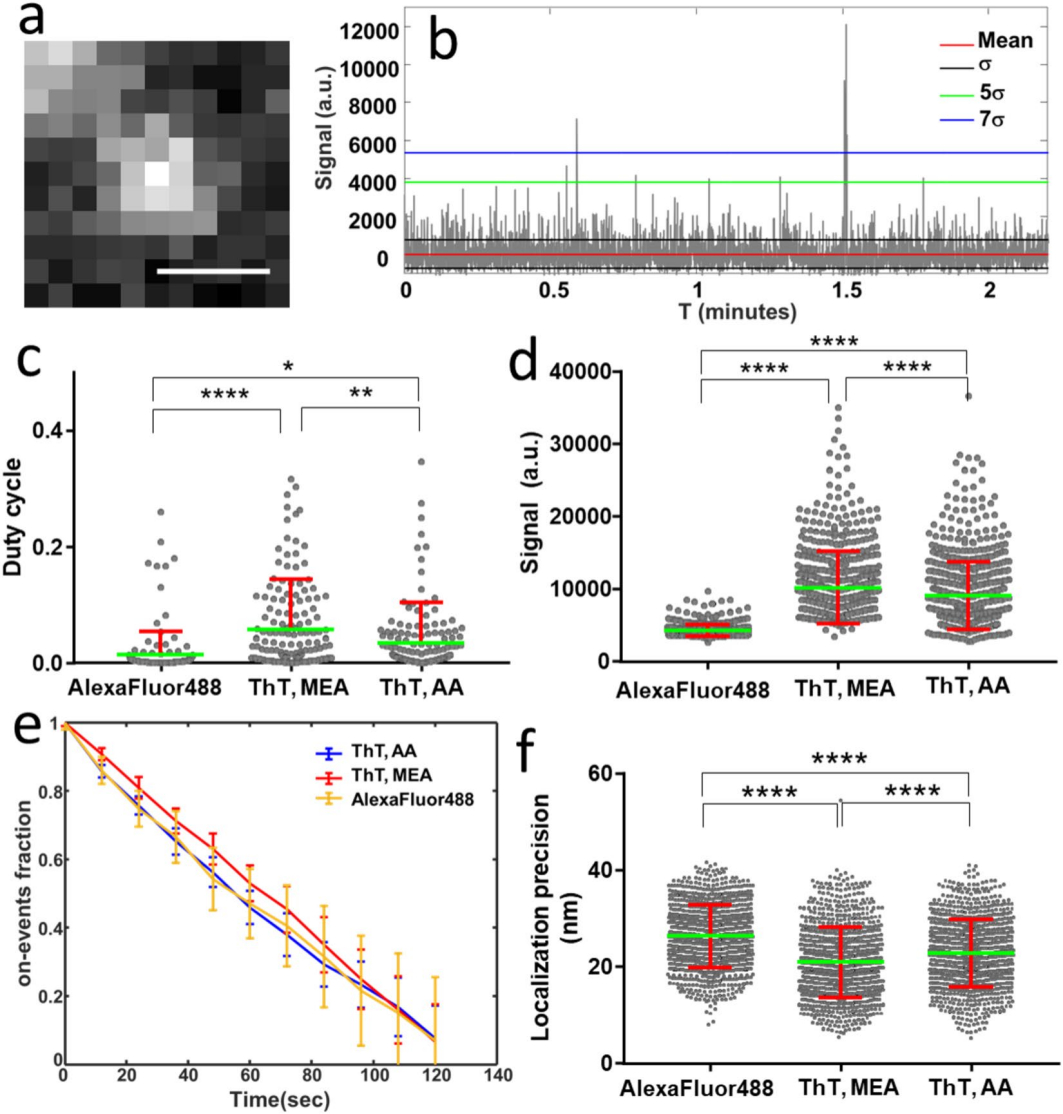
(**a**) Typical single ThT molecule image depicting the integration area used for the intensity time trace analysis. Scale bar: 500 nm. (**b**) Typical time trace (backround is removed) obtained at the location of a single ThT molecule (in MEA conditions), showing the standard deviation of the noise ${\rm{\sigma }}$, and the magnitude of a few ${\rm{\sigma }}$. (**c**) Average duty cycle obtained for the different conditions: ThT in MEA (n = 191), ThT in AA (n = 191), Alexa Fluor 488 in MEA (n = 155) deposited on a glass substrate (n: number of time traces over 6 different measurements, for each condition). (**d**) on-event signal (in camera counts) obtained for the different conditions: ThT in MEA (n = 774), ThT in AA (n = 774), and Alexa Fluor 488 in MEA (n = 774) (n: number of detected on-events, for each condition). The thresholding is $\,5{\rm{\sigma }}$. (**e**) Photostability duration of detected molecules (e.g. decrease of on-events over time measured from time traces), normalized to the total number of on-events and represented in a cumulative plot, averaged over 6 measurements per condition. (**f**) Localization precision deduced per detected single molecule in a dSTORM movie stack, for ThT in MEA (n = 1000), ThT in AA (n = 1000), and Alexa Fluor 488 in MEA (n = 1000). All error bars represent the standard deviation over n, number of measured samples.

Insulin amyloid fibrils were prepared with labeled ThT molecules and spin-coated onto a poly-L-lysine coated coverslip by rotating at low speed to get well separated fibrils of different orientations (see Methods). Upon strong illumination at 457.9 nm from a Ar-ion laser, a large portion of ThT molecules were seen to be sent into their dark state, with a visible on-off photo-switching behavior that permits one to identify single ThT molecules (Fig. 1a). To evaluate the ThT photo-switching properties from stack of images taken at 33 Hz rate, single molecules were isolated by a detection algorithm and their intensity deduced from an average within a 11 × 11 pixels detection window (pixel size 107 nm, see Supplementary Information). Note that molecules are visible over a slight diffuse background, which might be a residual fibrils autofluorescence. The background signal however does not strongly affect the single molecules detection in the algorithm. Time traces of single ThT molecules were recorded over a typical duration of a few minutes (Figs 1b and S1). On-events were extracted from the sequences by applying an intensity thresholding of typically five times the standard deviation of the background noise (Figs 1b and S2). From those on-events we calculated the averaged signal per on-event, the duty cycle of photo-switching, and the decrease of the number of on-events over time, which permits to quantify the molecules photostability. These quantities were compared with those of the widely used Alexa Fluor 488 molecule, directly deposited on a glass coverslip and in MEA buffer under its optimized reported dSTORM conditions^38^ (see Methods). The statistics on the measured quantities were performed on a collection of about 100–300 time traces per sample condition.

Using primary thiol MEA as a reducing agent, the obtained duty cycle of ThT is 0.056 ± 0.006 (Standard Error of the Mean, SEM), which decreases to 0.034 ± 0.005 (SEM) in AA as a reducing agent (Fig. 1c). In comparison to ThT, Alexa Fluor 488 in MEA shows an average duty cycle of 0.014 ± 0.003 (SEM), which is around three to four folds lower than ThT. The averaged signal per ThT molecule, obtained by averaging the direct recorded on-event signal (in camera counts) over the number of recorded molecules, is 10157 ± 180 (SEM) in MEA, and 9017 ± 166 (SEM) in AA, showing no strong differences (Fig. 1d). In comparison the average intensity obtained for Alexa Fluor 488 is 4241 ± 28 (SEM), which is more than two fold less than for ThT. Note that the data for ThT are generally more dispersed than for Alexa Fluor 488, which can be an indication of larger heterogeneity of local environments within the packing of amyloid β sheets. Both average duty cycle and on-event signal show that ThT is an acceptable photo-switching probe for stochastic super resolution imaging in both MEA and AA buffers, as compared to Alexa 488, which is one of the most effective dye for dSTORM in the blue-absorption range^31^. Note that other typical dSTORM dyes exhibit duty cycle values between 0.0001 and 0.04^31,39^, the lowest values being obtained in red-emitting cyanine derivatives that surpass oxazines and the rhodamine dyes. ThT is found to behave closer to rhodamine-like fluorophores.The reached duty cycle values for ThT are nevertheless not hampering dSTORM imaging to be achieved, as seen in other molecular examples^31^.

Note that ThT does not compare in structure to the dyes regularly used in dSTORM, however the factors necessary to favor photowitching and long off times are attributed to common properties of fluorophores, e.g. intersystem crossing and enlarged life time of their dark state(s). This off state prolongation is generally achieved via oxidation or reduction with the use of thiol or reducing agents^38^, depending on the possibility to induce efficient electron transfer in the system^37^. In this respect, the non negligible probability of ThT to relax to twisted states^40,41^, its intersystem crossing down to the triplet state^42^, and its potential electron affinity in its twisted state^41^, make it most probably a favorable route towards dark states control. Moreover, the duty cycle of ThT is seen to be slightly lower in AA, which could be attributed to the larger sensitivity of its benzothiazole group to reducing agents. This lower duty cycle in AA could in particular be exploited in imaging conditions with high labeling density. Indeed, the maximum number of fluorophores localized within a diffraction-limited volume is inversely proportional to the duty cycle, a high duty cycle thus leads to a requirement for reducing the number of accessible localizations, which ultimately affects the global resolution^31^. Second, the signal accessible per on-event is seen to be about 2.5 higher in ThT as compared with Alexa Fluor 488, with a slightly better efficiency in MEA. This is a prerequisite for high precision in the determination of single molecules’ positions, which follows an inverse-square-root dependence on the signal level, leading ultimately to higher resolution^32,33^.

At last the photostability of ThT is seen to be similar in both buffer conditions, and comparable to Alexa Fluor 488, which is a signature of a relatively stable behavior of the emission of single ThT molecules over time (Fig. 1e). Note that we observed a 5% increase of the number of molecules measured using an illumination in the UV range (405 nm, 250 ms, 0.1 kW/cm^2^) (see Supplementary Information), indicating a slight advantage in using UV light as a reactivation processes, with similar efficiency as reported for part of the dSTORM dyes absorbing in the blue and yellow regions^38^. This sensitivity of ThT to UV could be attributed to the fluorescence recovery of UV-absorbing twisted conformations of ThT that are more favorable to some of the amyloids binding sites^43^, which would necessitate photophysical studies to be fully understood.

Finally in order to relate the properties measured for ThT to practical dSTORM imaging conditions, we deduced the obtained localization precision and signal to noise ratio (SNR) for each single ThT molecule, using a detection algorithm derived from Multiple-target tracing algorithm (MTT)^44^ (see Supplementary Information). For a similar labeling density, the obtained averaged localization precision is 20.91 ± 0.23 (SEM) nm for ThT molecules in MEA, 22.8 ± 0.22 (SEM) nm in AA, and 26.31 ± 0.20 (SEM) nm for Alexa Fluor 488 (Fig. 1f).

**Figure 2:**
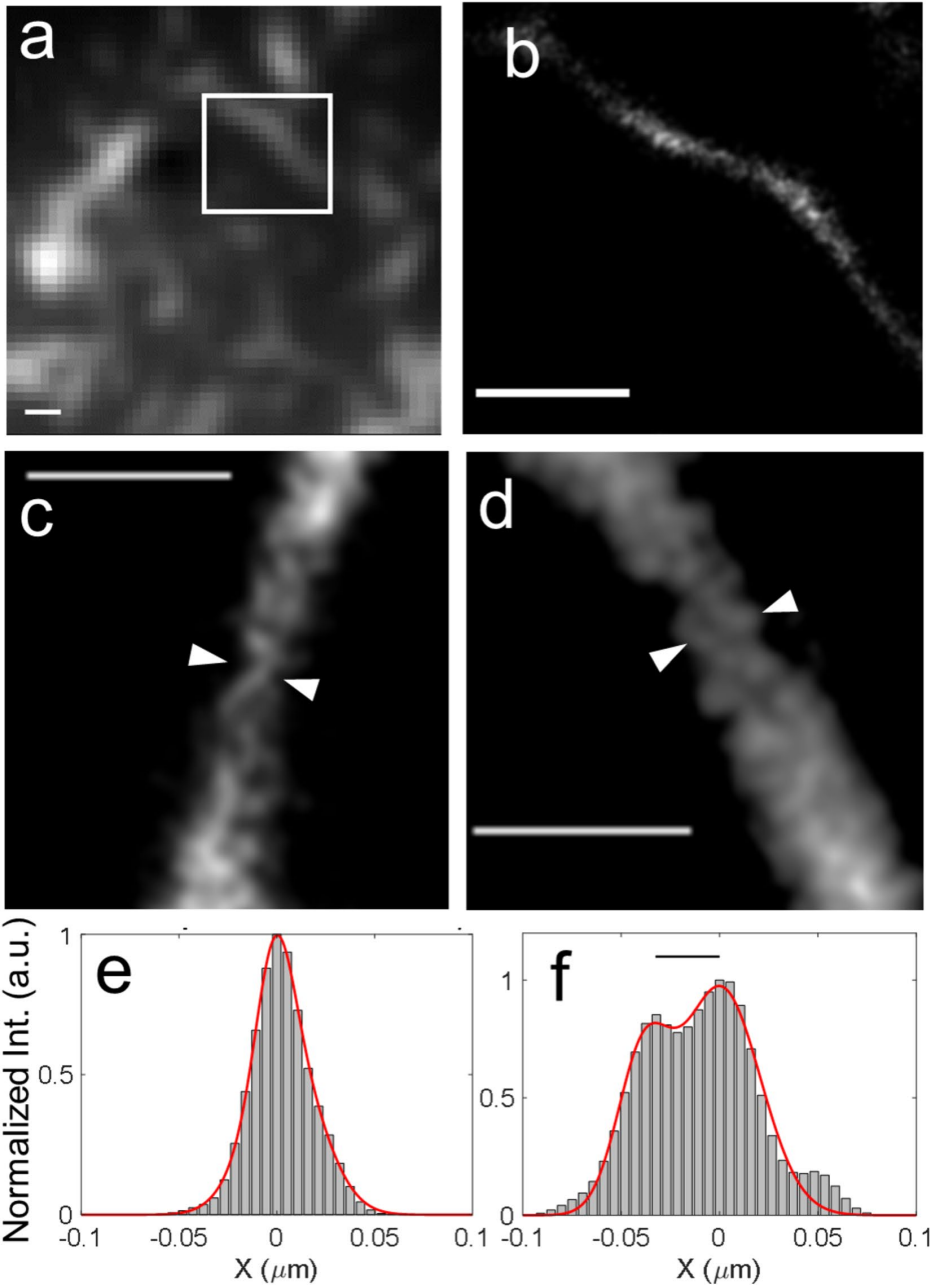
(**a**) Wide field imaging of insulin amyloid fibrils labeled with ThT in AA. Scale bar: 500 nm. (**b**) dSTORM image of a zoomed part of image (**a**) (shown by a white square). Scale bar: 500 nm. Gaussian blur: 20 nm. (**c**) dSTORM zoomed image of a fibril in MEA. Scale bar: 200 nm. (**d**) Similar dSTORM image zoom, using the switching buffer AA. Scale bar: 200 nm. (**e**) Profile of image (**c**) (showing molecules positions between white arrows) and corresponding fit by a Gaussian curve. Full Width Half Maximum (FWHM): 21.9 nm. (**f**) Similar approach for the profile shown in image (**d**). A two Gaussian fit is shown, with interdistance (black line) of 32.5 nm.

These values support the use of ThT for super-resolution imaging, and confirm the effect of its higher efficiency in number of photons detected per molecule. The consequence of high signals measured is also a high SNR for ThT, which is favorable for dSTORM reconstruction (see Supplementary Figure S3). dSTORM reconstructions of amyloids labeled with ThT molecules used typically 10 000 frames (see Supplementary Information). The results, shown in Fig. 2 for both MEA and AA conditions, emphasize the large gain in resolution as compared to wide field imaging for both buffers, with similar performances.

dSTORM images show elongated thin filaments, within which a close view frequently exhibits a helical-like structure (Fig. 2c,d). Noticeably, the difference of duty cycle values for the two tested buffers did not seem to strongly affect the quality of the obtained images, showing that the reconstruction process is also largely governed by the intensity per molecule, which is very high for ThT in both buffers. At the level of density used and number of images, the difference in duty cycle did not induce a strong difference in image reconstruction.

We estimated the imaging resolution by fitting profiles obtained along the different regions of the obtained fibrils. Typical fibril diameters lie from 22 nm to 80 nm (Fig. 2e), with a lower limit close to the localization precision. This resolution, which is similar to that obtained in super-resolution images using covalent labeling of amyloids^16^, represents a considerable gain as compared to diffraction limited wide field microscopy. Interestingly, the morphological properties of helical-like structures within some portion of large fibrils could be quantified, with evidence of twisted lines with an inter-distance of typically 30–35 nm (Fig. 2f). Amyloid fibrils, among them the widely used insulin, are known to be made of twisted ribbons formed from interlaced individual 3–4 nm diameter filaments. Depending on the number of filaments involved, helical structures exhibit a diameter 7–16 nm and pitch length ranging from 35 to 95 nm^9,45^. This variability in morphology is essentially due to slight modifications in the β-sheet twist within single filaments^45,46^. In the dSTORM images obtained, the typical apparent size of the helical pitch is about 100 nm, which lies close to the structure obtained from large fibrils. The present method appears thus as a possible way to decipher filaments morphology within large amyloids fibrils.

**Figure 3:**
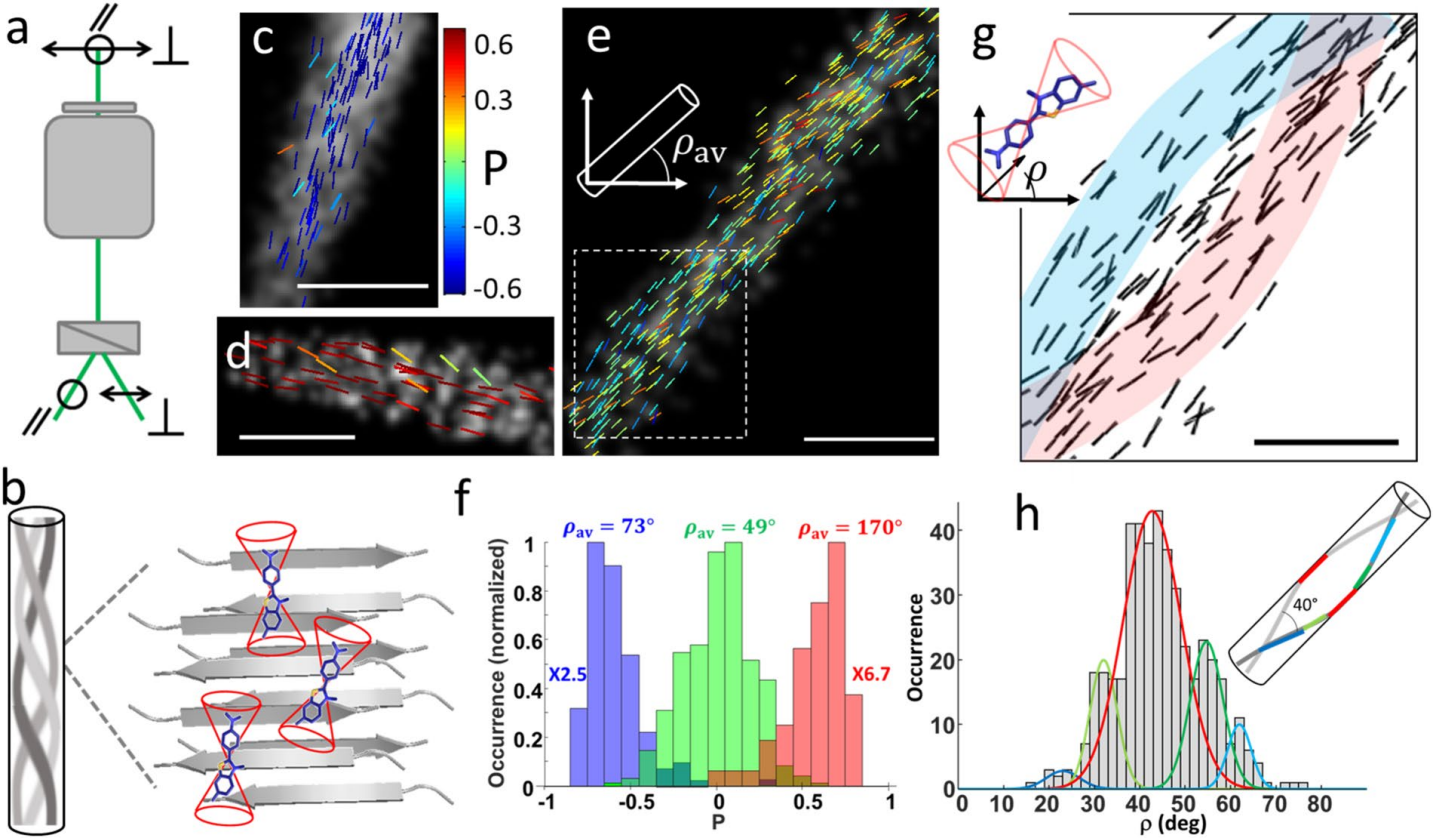
Polar dSTORM imaging of insulin amyloid labeled with ThT. (**a**) Principle of P-dSTORM, showing horizontal and vertical polarization directions collected. (**b**) Schematic representation of a twisted ribbon made of three protofilaments inspired from measured structures^9,45^. The zoomed view (within a full field of view of typically 20 μm × 20 μm) shows β-sheets embedding ThT molecules which undergo orientational wobbling within a cone of aperture δ. (**c**–**e**) dSTORM image of ThT in amyloid fibril regions showing in superposition the result of polarized analysis. Sticks are colored with their *P* values and their orientation follows the *ρ* orientation relative to the horizontal direction, for a vertical (**c**), horizontal (**d**) and 45° oriented fibril (**e**). Not all molecules detected by dSTORM are represented in P-dSTORM, due to the more stringent intensity thresholding that is imposed to retrieve high precision *P* (here 0.08)^25^. Scale bars: 200 nm. (**f**) Histogram of *P* values measured on the fibrils shown in (**c**–**e**), together with their mean orientations ${\rho }_{av}$ measured from the dSTORM image. (**g**) Same fibril as in (**e**) (zoom on dashed rectangle) where only *ρ* is represented as black sticks to evidence preferential directions in the fibrils, shown schematically as colored guide lines. Scale bar: 100 nm. (**h**) Histogram of measured *ρ* orientations within the fibril portion in (**g**), evidencing populations of preferred directions which are fitted with a Gaussian distribution. The parameters of the Gaussian fits are (in increasing *ρ* order, giving their center and full width half maximum): 23°(FWHM 8°), 32°(7°), 43°(15°), 55°(8°), 62°(6°). The measured orientations are shown superimposed with a twisted helical geometry with a 4$0^\circ$ angular aperture.

In order to further investigate organization within a single amyloid fibril, we combined pure dSTORM imaging with the detection of the polarized nature of ThT molecules emission, which is related to their orientation. Ideally monitoring ThT orientation should allow following protofilaments directions within the fibrils, giving unambiguous access to their ultrastructure. In particular the 2D projection of a twisted ribbon should lead to preferred in-plane orientations along specific directions. Several methods have been reported to retrieve single molecule orientations, using defocusing imaging strategies^47,48^, or alternatively a modification of the emission Fourier plane of the microscope^49^ or polarization splitting at the excitation and/or detection steps^50,51^. Only a few methods have however achieved the capacity to determine both orientation and localization information from single molecules, which is a pre-requisite for orientation-sensitive dSTORM imaging^52^. A recent work has used sequential illumination by three polarization states combined with super-resolution localization imaging^27^. This method however requires the fluorophores to be present in a few consecutive images, which is delicate to guaranty in the most general dSTORM conditions. We rather opted for the splitting of single images into two polarized images, in a method named polarized dSTORM (P-dSTORM) imaging^25^, which allows direct determination of polarized information from a single frame. Note that both methods are limited to the detection of molecules lying majorly in the sample plane and are thus intrinsically 2D, which provides nevertheless a large amount of information for structural analyzes. The principle of P-dSTORM is to split the images obtained in a dSTORM sequence in two sub-images polarized perpendicularly to each other (named || and $\perp$ directions), measuring two intensities, ${I}_{\parallel }$ and ${I}_{\perp }$ (Fig. 3a) (see Methods and Supplementary Figure S4). The use of low tilt angle illumination and a numerical aperture below the critical angle conditions moreover ensure sufficient preferential in-plane molecules excitation and detection to avoid bias on P values^25^.

By computing a ratiometric polarization factor $P=({I}_{\parallel }-{I}_{\perp })/({I}_{\parallel }+{I}_{\perp })$ for molecules on a fibril of known direction, it is possible to extract their mean orientation and orientational flexibility (wobbling), in 2D, providing the hypothesis that the wobbling angle is the same for all molecules^25^. Figure 3c–e show dSTORM images (grey background) on which molecules are represented as sticks, which color encodes the measured polarization factor, and which orientation shows directly the molecule mean orientation using a methodology detailed below and in the Supplementary Information. $P$ values clearly depend on the fibril orientation (named ${\rho }_{av}$ in Fig. 3e,f), with highest $|P|\,\,$values for horizontal and vertical fibrils, and $P \sim 0$ for fibrils oriented at 45°. This behavior is the signature of alignment of ThT molecules along the fibrils (see Supplementary Figure S5). The extreme values reached by $|P|\,\,$for vertical or horizontal fibrils (Fig. 3c,d,f) are however not $\pm 1$, as would be expected from fixed molecules (see Supplementary Information). This evidences a non-negligible single molecule wobbling, which can be quantified using the methodology developed in ref.^25^ that plots the measured $P$ values on a theoretical $P(\rho ,\delta )$ curve for fibrils of known macroscopic directions (see Supplementary Information). Plotting several measured fibrils or different orientations leads to a lower bound value for the wobbling angle of about $\delta  \sim$60° (see Supplementary Figure S6). Note that since the method cannot decipher independently $\rho$ and $\delta$ values, this $\delta$ value is assumed to be the same for all measured molecules. Nevertheless despite this approximation, the result shows unambiguously a wobbling of ThT molecules in the β-sheets of amyloid fibrils, within the 30 ms measurement integration time. Angular fluctuations might not be expected from the tight ThT binding to the amido acid sequences^23,53,54^. Interactions of ThT molecules within the amyloids binding sites are however reported to be heterogeneous in nature, due to steric hindrance, aromatic and hydrophobic interactions, as well as electronic stabilization. While the exact mechanism of ThT stabilization and its consequence on the high fluorescence signal is still under study^41^, molecular dynamics simulations have reported a non negligible degree of orientational freedom for ThT bound to amyloids^54,55^.

The measured flexibility $\delta$ value also evidences that the orientational disorder previously measured in ensemble measurements in ThT bound amyloids is in large portion attributed to molecular fluctuations^8^. Reported ensemble disorder values range between 90° and 120°^6,8^, which encompass both intrinsic fluctuations of ThT molecules and structural disorder of fibrils at sub-diffraction scales. These values corrected for the molecular wobbling obtained here by P-dSTORM would lead to a structural disorder in the range 30°–60°. In P-dSTORM it is possible to map this structural disorder at the single molecule scale (Fig. 3g). To obtain this structural map, we corrected each single molecule orientation data by the molecular wobbling value, retrieving its orientation *ρ* by the use of the $P({\rm{\rho }},\delta )\,\,$dependence when $\delta$ is known (see Supplementary Information). With the thresholding imposed on the molecules intensities, the precision on *ρ* is expected to be of the order of 4°, as simulated from noise^25^. The result obtained in Fig. 3g shows that mean molecular directions are not randomly distributed but preferentially follow specific directions within a pattern which resemble locally a twisted structure, similarly to the shape observed in pure dSTORM imaging. Here the information is however directly obtained from molecules’ orientation, rather than from a morphological image, thus reporting directly local structural information. In particular, the mean orientations of single ThT molecules within the measured fibrils are seen to explore only a few preferential directions (Fig. 3h), which is characteristic of the existence of a long-pitch helical structure. Note that the experiment intrinsically filters such preferential directions due to its 2D nature, the P-STORM scheme being essentially sensitive to in-plane molecules^25^. From the measured directions, a helical pitch angle can be deduced to be about 40° (Fig. 3h), with variations between fibrils from 20° to 40° (see Supplementary Figure S7). This value is in agreement with high order helical amyloid structures previously reported in thick filaments by electron microscopy and atomic force microscopy^9,45^. Comparing these data with the previously reported ensemble measurements, the addition of both wobbling effect and this structural disorder leads to average values close to the reported ensemble disorder^6,8^.

Note that the single molecule P-dSTORM method could also be sensitive to the heterogeneity of ThT binding conformations. Several binding sites have indeed been reported within amyloids, which depend on the local amino acid sequence, which could also correspond to different orientations of the dye^45^. Dynamics spectroscopy studies have however shown that among the most probable binding sites at the inner-channel or at the surface of the β-sheets, the inner-channel positions correspond to the highest fluorescence quantum yield and shortest life time^56^. Considering the high intensity thresholding imposed for the single molecules intensities in P-dSTORM, the most probable population observed is therefore likely to be the inner-channel one, which potentially reports fibrils directions with high fidelity when corrected for wobbling. At last, even though this measurement might not be sensitive enough to reveal all hierarchical helical sub-structures present within the filaments assemblies, in particular at smaller single-filament scales^9,45^, it reveals a predominant high order structure along the fibril which confirms the morphological properties of protofilaments within amyloids. This information, which is obtained using single molecule information, would not be accessible using ensemble polarized measurements.

To conclude, measurements of photo-switching properties have shown that ThT is a good candidate molecular probe for stochastic super resolution imaging. Most importantly, it is appropriate for P-dSTORM imaging, which provides ultra-structure analysis of filaments assemblies within amyloid fibrils. The possibility to access such information could be ultimately used for addressing open questions on the growth mechanism of amyloids, the level of protofilaments assemblies being yet not reachable despite the use of super resolution imaging.

## Methods

### Cleaning and preparation of coverslips and slides

Coverslips and slides were cleaned with Acetone-ddH2O-methanol- ddH2O (five times, three minutes each time) under running flow. Then, coverslips were sonicated in 1 M KOH for 30 minutes. Coverslips were thoroughly rinsed under running water followed by immersion in acetone for 10 min. Before use, they were extensively rinsed under water, and blown dry. Cleaned coverslips were coated with poly-L-lysine (PLL; MW 70000-150000, Sigma) by adsorption from a filtered 0.01% w/v solution for 1 hour, carefully rinsed with water and blown dry.

### Amyloids samples preparation

Bovine insulin powder and Thioflavine-T were purchased from Sigma. Insulin was dissolved at a concentration of 2 mg/mL in acid buffer (pH = 2, 20% acetic acid, 100 mM NaCl), then sonicated for 5 min to dissolve aggregates. Fibrillation process was induced by heating the insulin solution at 50 °C for 20 h without agitation. The solution was cooled down at room temperature. Slide-A-Lyzer G2 Dialysis Cassettes (Thermo scientific) was used for removal of insulin monomers. For several experiments, insulin fibrils were further concentrated by removal of supernatant after centrifugation at 27 000 × *g* for 30 min. The ThT solution was diluted to a 1 μM final concentration. Equal volumes of ThT and insulin fibrils were mixed to get labeling ratio 1:20 (dye/monomer)^57^. The final solution of labeled amyloid-ThT was adsorbed to the coverslip and dried in vacuum chamber at room temperature. Coverslips with labeled amyloid-ThT and glass slides were then attached to a Cover Well imaging chamber (Grace Bio Labs), which contained the photo-switching buffer. Two buffers were tested, in the presence of an enzymatic oxygen scavenging system (0.5 mg/ml glucose oxidase, 40 μg/ml catalase, 10% w/v glucose): either the primary thiol mercaptoethylamine (MEA), or the reducing agent ascorbic acid (AA) were used. MEA was stored at 4 °C. Before the experiment, 50–100 mM final thiol- concentrations were prepared in PBS (no significant difference in photoswitching properties were found for this concentration range), with final pH ~ 8 adjusted with KOH. Fresh AA solution of concentration 10–100 µM was prepared in phosphate-buffered saline (PBS).

### Single Alexa Flour 488 molecules preparation

The stock solution of Alexa 488 was diluted to 1 μM as final concentration and spread on coverslips, then incubated in vacuum chamber at room temperature to let it dry. The Alexa Flour 488 molecules were then adsorbed on the glass slide in a cover well imaging chamber (Grace Bio Labs), which contained a switching buffer known to be optimized for this dye^38^: MEA (100 mM at pH ~ 8) and oxygen scavenging system.

### dSTORM and P-dSTORM imaging

The experimental setup is based on an epi-fluorescence wide field microscope which follows the principle of dSTORM. The excitation light source is a polarized continuous 457.9 nm Ar-ion laser line (Coherent), with typical averaged power density of about 3–5 kW/cm^2^ before the objective (5 kW/cm^2^ was used for both ThT and Alexa Fluor 488 for their photoswitching properties comparison, as fixed from Alexa Fluor 488 known optimal conditions^38^). The laser beam is expanded by a telescope and circularly polarized by quarter waveplates (WPQ10M, Thorlabs), reflected on a dichroic mirror (Di02-R488-25 × 36, Semrock) with excitation filter (FF02-475/50-25, Semrock), and focused by a large focal length lens (f = 400 mm) in the back focal plane of the objective (Plan Apo 100x, NA = 1.49, Nikon). This provides a wide-field illumination of diameter of about 100 μm in the sample plane, which is slightly tilted to reduce fluorescence background from the solution. The emitted fluorescence is collected by the same objective lens in an epi-geometry, passes through the dichroic mirror and a band pass emission filter (FF01-535/675, Semrock). The imaging lens produces an image with a total magnification of 150 x on a EMCCD camera (Quantem 512sc, Photometrics). For polarized dSTORM imaging (P-dSTORM), experimental conditions reduce the detection to mostly in-plane molecules, by exciting the sample under low tilted illumination and using a reasonably low aperture objective (Plan Apo 60x, NA = 1.3, Nikon). This geometry considerably reduces biased detection due to out of plane orientations^25^. The detection beam passes a Wollaston prism (separation angle 5°, CVI Laser Optics) placed just before the EMCCD camera to fit within the camera chip two images, from the perpendicular (“⊥”, corresponding to the vertical axis of the sample plane) and parallel (“∥” corresponding to the horizontal axis of the sample plane) polarization states. The size of the images is set by a diaphragm placed at the first image plane at the exit of the microscope. The polarization homogeneity the field of view is seen to be satisfactory, as measured using a rhodamine 6 G water solution which also serves polarization factors calibration (see Supplementary Information). P-dSTORM image processing relies on the common detection and localization of each single molecule on both polarization states (⊥ and ∥), plotting for each of them the polarization factor *P* (see Supplementary Information). Molecules for which the intensity was too low (below 20 000 camera counts) were ruled out of the analysis, in order to ensure a high precision (better than 0.08) for the determination of *P*. Interpretation of the polarization factor into orientation information followed the procedure described in ref.^25^ (see Supplementary Information).

### Statistical analyses

All statistical analyses were performed using the Prism 6.0 software (GraphPad Inc.). For comparing more than two conditions, one-way ANOVA tests were used. Significance of mean comparison is marked on the graphs by asterisks with (*$p\,\le 0.05$) considered as statistically significant; (**$p\,\le 0.01$) as highly statistically significant; (***$p\,\le 0.001$) and (****$p\,\le 0.0001$) as extremely statistically significant.

### Data availability

The datasets generated and analysed during the current study are available from the corresponding author on reasonable request.

## Electronic supplementary material

Supplementary Information
