# Supplementary material for: Polarized super-resolution structural imaging inside amyloid fibrils using Thioflavine T
Source: Sci Rep. 2017 Oct 2;7:12482. doi: 10.1038/s41598-017-12864-9 (PMC5624930; doi:10.1038/s41598-017-12864-9)
Supplement: Supplementary file 1 — Supplementary Information [file 41598_2017_12864_MOESM1_ESM.pdf]

# Supplementary Information

# Polarized super-resolution structural imaging inside amyloid fibrils using Thioflavine T

Haitham A. Shaban[†,‡], Cesar A. Valades-Cruz[&,‡], Julien Savatier and Sophie Brasselet[*]

Aix Marseille Univ, CNRS, Centrale Marseille, Institut Fresnel, F-13013 Marseille, France

[†] Present address: Laboratoire de Biologie Moléculaire Eucaryote (LBME); Centre de Biologie Intégrative (CBI); Université de Toulouse; CNRS; UPS; 31062 Toulouse, France, and Spectroscopy Department, National Research Centre, El-Bohouth Str., 12622, Dokki, Giza, Egypt.

[&] Present address: Institut Curie - Centre de Recherche, PSL Research University, Endocytic Trafficking and Intracellular Delivery Laboratory, 75248 Paris cedex 05, France.

[‡] These authors contributed equally to the work.

[*] sophie.brasselet@fresnel.fr

## Content:

### Supplementary Notes

1. **dSTORM data processing and analysis.**

   This section contains details for: Single molecule localization and dSTORM imaging; Analysis of single-molecule time traces; Duty cycle; Photostability; Localization precision; Sensitivity of ThT to ultra violet light activation.

2. **P-dSTORM image processing and analysis**

### Supplementary Figures

**Figure S1.** Typical single molecule fluorescence time traces of ThT bound to insulin amyloid fibrils.

**Figure S2.** Dependence of the parameters duty cycle, total number of molecules, signal (in camera counts) deduced from different settings of data detection and thresholding of the time traces measured.

**Figure S3.** Localization precision and dSTORM condition quality.

**Figure S4.** P-dSTORM imaging on ThT bound to insulin amyloid fibrils.

**Figure S5**. Ensemble polarized information gained from P-dSTORM imaging.

**Figure S6.** Molecular order probed by P-dSTORM single molecule information.

**Figure S7.** P-dSTORM data on a single ThT bound insulin amyloid fibril portion.

## Supplementary Notes

### 1. dSTORM data processing and analysis.

**Single molecule localization and dSTORM imaging.** Single molecule localization and dSTORM reconstruction were obtained using custom scripts written in Matlab®, based on Multiple-target tracing algorithm (MTT)[1]. In a first detection step, a Generalized likelihood ratio test (GLRT) is used to identify the single molecule candidates for dSTORM. During this detection step, one fixed Gaussian shape is assumed for the theoretical PSF (radius 1.3 pixels within a detection window of 11 x 11 pixels, pixel size 107 nm), as detailed in the original MTT algorithm[1]. Then the amplitude, radius and position of the Gaussian PSFs are estimated for the candidates at the subpixel scale, based on a Maximum likelihood (ML) estimation using a Gauss-Newton regression. This regression uses the GRLT obtained values of radius and position as initial parameters. The localization accuracy is estimated from a computation of the Cramer-Rao bound (CRB) limit. Two post-processing steps are then performed. First, low density localizations are removed, by filtering out events which contain below 3 localizations identified in a region of size 50 nm × 50 nm. Second, lateral drift is corrected using a cross-correlation procedure similarly as in[2].

**Analysis of single-molecule time traces.** For single molecule traces analysis, movies of 5000 frames at 33 Hz frame rate were recorded. 6 movies per experiment condition were analyzed (THT+MEA, THT+AA and Alexa Fluor 488). The excitation power to switch most of the single molecule dye into a dark state was ~3-5 kW/cm$^2$. Time traces analysis was performed by a custom Matlab script. First, candidate molecules were detected every 1000 frames using the detection algorithm described previously for the dSTORM imaging. Only isolated molecules were selected, meaning a molecule that is not located within the distance from other corresponding to 4 pixels. Then a time trace per molecule was defined as a 150 s duration frame sequence measuring the signal from the detected molecule (Figure S1). In total, 100-300 time traces could be retrieved for each sample condition. Within these time traces, the molecule signal per frame was defined as the integrated signal over a 11×11 pixel window after subtracting pixel background. Pixel background was defined as the average of the pixels surrounding this detection window. Time traces were analyzed based on different thresholding levels, from 5 to 10 times the standard deviation of the noise ($\sigma$) during the full time trace (Figures S1, S2).

**Duty cycle.** The $\mathrm{on/off}$ duty cycle is a crucial property for switching fluorophores, in particular it scales as inversely proportional to the maximum detectable number of fluorophores in the diffraction-limit spot[3]. As a consequence it limits the expected image

resolution since based on the Nyquist criterion (the image sampling interval must be smaller than half the desired resolution), the resolution is twofold the distance between nearest fluorophores[3,4]. Using the retrieved time traces described above, the duty cycle was estimated per single-molecule time trace using a custom Matlab Script, using the “dutycycle” command from the Signal Processing Toolbox. Molecule signals were considered “on”, when the signal was higher than 5σ (Figure S1). Variations of the duty cycle value as a function of signal thresholding level are showed in Figure S2 for the different sample types studied. Around the threshold of 5σ the duty cycle is seen to be quite stable, which guaranties its robust statistical determination.

**Photostability.** In order to evaluate the fluorescence photostability properties of the studied molecules, the durations of the time traces described above were estimated using a threshold of 5σ, starting at the first measurement point considered as time t = 0. For this, the number of on-events averaged over 400 frames, was calculated every 400 frames for the whole time trace duration (5000 frames). This number was then normalized to the total number of on-events and represented in a cumulative plot as a function of time. For each measured condition, the plot was averaged over 6 measurements.

**Localization precision.** For the estimation of the localization precision and standard deviation of the noise (σ) per molecules, 6 movies per experiment condition were analyzed (THT+MEA, THT+AA and Alexa Fluor 488). The localization precision was estimated from a computation of the Cramer-Rao bound (CRB) limit, described in[1]. Single molecules localizations, amplitudes ($\alpha$) and PSF radius (*r*) were obtained from the Maximum likelihood (ML) estimation using a Gauss-Newton regression (Figure S3)[1]. Intensities were obtained from the integration of the obtained amplitude over the PSF size. Standard deviation of the noise (σ) and signal to noise ratio (SNR, defined as the ratio of a signal (square amplitude $\alpha^2$) to the noise (variance $\sigma^2$) corrupting the signal), were obtained from the regression, as described in the MTT algorithm[1].

**Sensitivity of ThT to ultra violet light activation.** The photobleaching of fluorophores is caused by light excitation in the presence of molecular oxygen, which ultimately causes irreversible damage to the fluorophore due to chemical reactions with its de-excitation states[5]. Photobleaching of fluorophores affects negatively the number of detectable molecules through the acquisition duration. The decrease of detectable molecules leads ultimately to an increase of the acquisition time required for reconstruction of a dSTORM image. It has been reported that using an additional excitation light at 405 nm can increase the time traces duration of most of the conjugated STORM dyes by a recovery mechanism through their excited state[4]. The percentage of recovered molecules upon

405 nm illumination depends on the spectral properties of the dyes, the recovery fraction of red and near infra-red colored-dyes being significantly increased by up to 30% due to 405 nm intermittent illumination[6]. We tested the sensitivity of ThT to ultra-violet-light activation in 5 samples under MEA buffer condition, by irradiating molecules in the field of view with a short duration (250 ms) of 405 nm light at 20 mW (OBIS, Coherent). A movie (5000 frames at 33 Hz frame rate) per sample of ThT-labeled amyloid was recorded in the same condition as for dSTORM imaging. The number of detected molecules recovered after the 405 nm laser illumination was compared to the initial number of molecules, using the recovery fraction $r = (\mathrm{N}_1 - N_0)/N_0$ , with $\mathrm{N}_1$ the average number of molecules per frame within the 100 frames after the application of the 405 nm laser illumination, and $\mathrm{N}_0$ is the average number of molecules per frame within the 100 frames preceding the application of the 405 nm laser illumination (10 to 20 molecules were detected per frame). A recovery fraction of $r \sim$ 5% was obtained due to UV illumination, on average over the 5 samples studied, indicating an effect of UV light activation[4]. This effect is an indication that absorption of UV light by ThT molecules might induce photo-oxidation of radical anions[6].

## 2. P-dSTORM image processing and analysis

Polarized dSTORM (P-dSTORM) processing was performed using a custom Matlab script described in[2], which consists in treating simultaneously the two detected polarized channels, in the ∥ and ⊥ polarization directions (Figure S4). This algorithm retrieves, per molecule, its position coordinates [($i_\parallel$ , $i_\perp$), ($j_\parallel$ , $j_\perp$)], radius of PSF ($r_\parallel$, $r_\perp$), and amplitude of the PSF ($\alpha_\parallel$, $\alpha_\perp$). This serves to calculate its intensities ($I_\parallel$, $I_\perp$) (amplitude integrated over the PSF) and finally its polarization factor $P = (I_\parallel - I_\perp)/(I_\parallel + I_\perp)$, and assign it a position. This polarization factor accounts for possible polarization distortions in the detection path of the microscope (induced by the dichroic mirror and other optics). For this, a calibration evaluates the diattenuation factor of the system by the use of a solution of rhodamine 6G fluorophores freely diffusing/rotating in water, which is expected to lead to a completely depolarized emission. The determination of the compensation factor G between the ∥ and ⊥ channels lead to G = 1.2, which is included in the estimation of $P$: $P = (I_\parallel - GI_\perp)/(I_\parallel + GI_\perp)$.

Note that only molecules for which the intensity is above a given value are treated (e.g. here 20 000 camera counts), which ensures a precision of better than 0.08 in the determination of $P$, which corresponds to angular precision of a few degrees for the determination of orientations of individual molecules). In order to determine from $P$ the

mean orientation ($\rho$) and angular fluctuation extent ($\delta$) experienced by the molecule during the integration time (30 ms) of P-dSTORM imaging, portions of amyloid fibrils of known orientation (from the wide field image) were individually studied. First, the averaged $P$ value is seen to depend on the fibril orientation in a way that indicates a non-negligible oriental order, similar to what is reported in other works[7], which validates the approach (Figure S5). A model supposing that molecules are wobbling within a cone of aperture $\delta$ that lies in the sample plane, leads to a known dependence $P(\rho, \delta)$ that can be exploited for analysis[2] (Figure S6). From this dependence, $\delta$ can be deduced from the extreme values reached by $P$, e.g. for either horizontal or vertical fibrils. From the collection of about 15 fibrils measured, a lower limit of $\delta \sim 60°$ could be inferred. Based on this $\delta$ value, the data obtained from individual fibrils of known orientations could be reported in the dependence $P(\rho, \delta = 60°)$ to retrieve the mean $\rho$ orientation experienced by each single molecule. This $\rho$ angle is reported on the dSTORM image as a stick orientation with angle $\rho$ relative to the horizontal direction, which color is the measured $P$ value. This allows in particular to visualize possible heterogeneities in $\rho$ orientations that would correspond to orientational disorder or to specific sub-fibrils (filaments) orientations (Figure S7). The total field of view of the sample accessible in P-dSTORM imaging is about 20 μm x 20 μm, which is governed by the magnification used and the splitting of the image plane.

**Supplementary Figures**

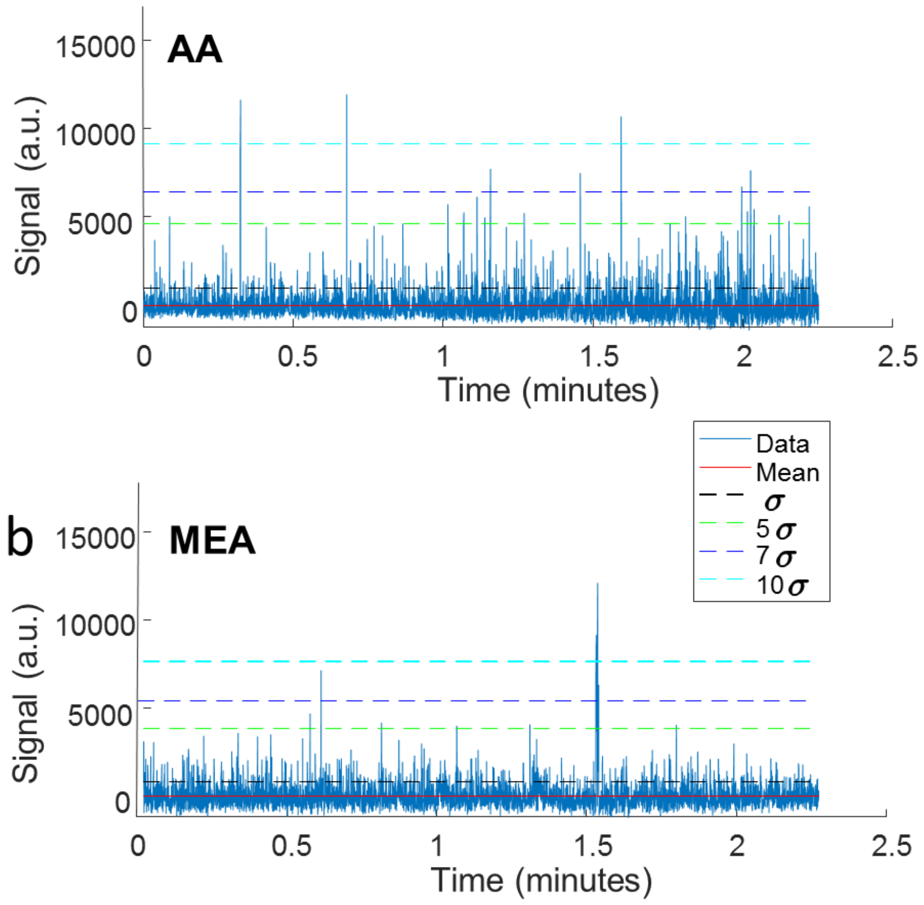


**Figure S1.** Typical single molecule fluorescence time traces of ThT bound to insulin amyloid fibrils in (a) ascorbic acid (AA) and (b) mercaptoethylamine (MEA), in the presence of an oxygen scavenging system. The signal level is given in camera counts. The dashed lines represent the measured standard deviation of noise ($\sigma$) and level reached at $5\sigma$, $7\sigma$, and $10\sigma$.

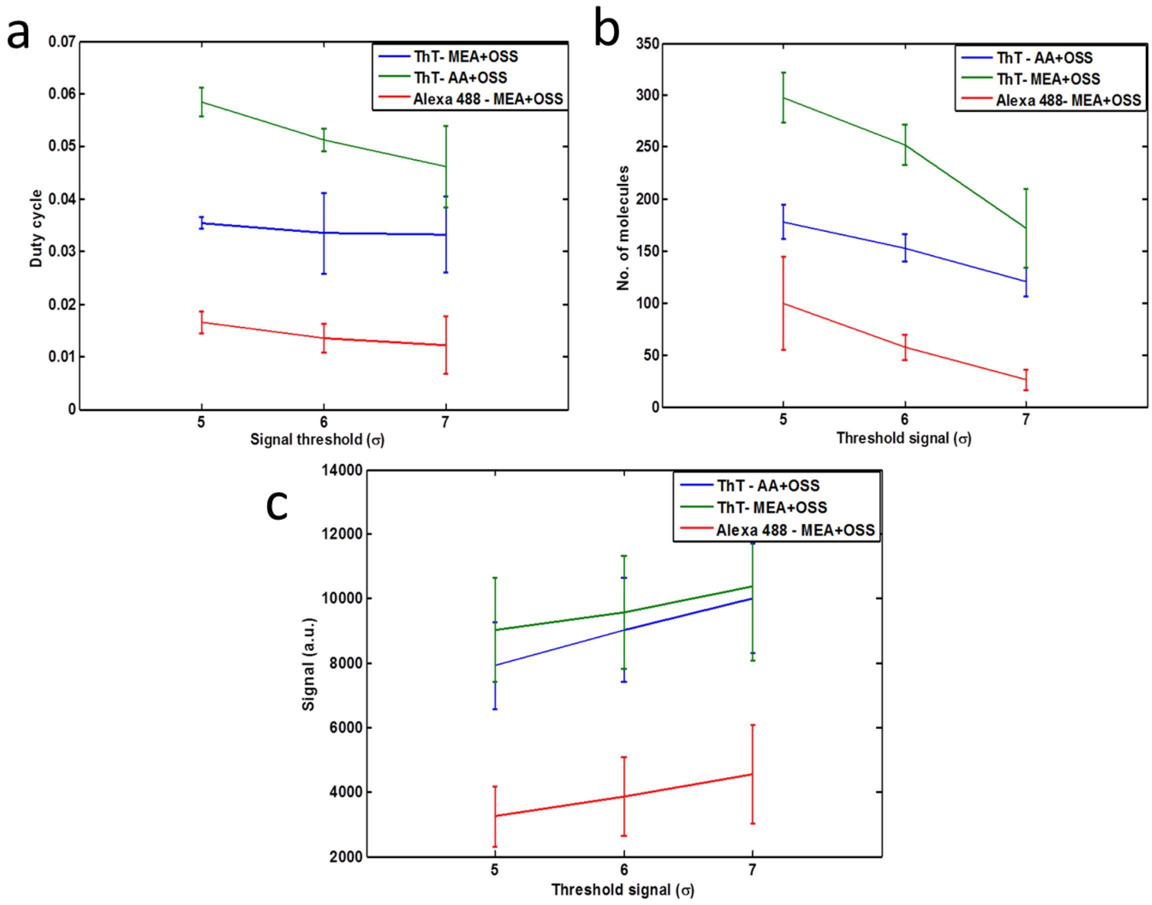


**Figure S2.** Dependence of the parameters duty cycle, total number of molecules, signal (in camera counts) deduced from different settings of data detection and thresholding of the time traces measured: 5σ, 6σ, 7σ (with σ the standard deviation of the noise). The data are averaged for different detection settings (Probability of False Alarm[1] (PFA values of 24 and 28 are used) and detection window (7 x 7 and 11 x 11 pixels are used). The error bars represent the standard deviation over the measured population. The parameters are measured for ThT bound to amyloids, in the two photo-switching buffers MEA and AA, as well as for Alexa Fluor 488 deposited on a coverslip in MEA. The measured duty cycles is seen to be only slightly dependent on the thresholding, even though the number of molecules is reduced by about 1.5.

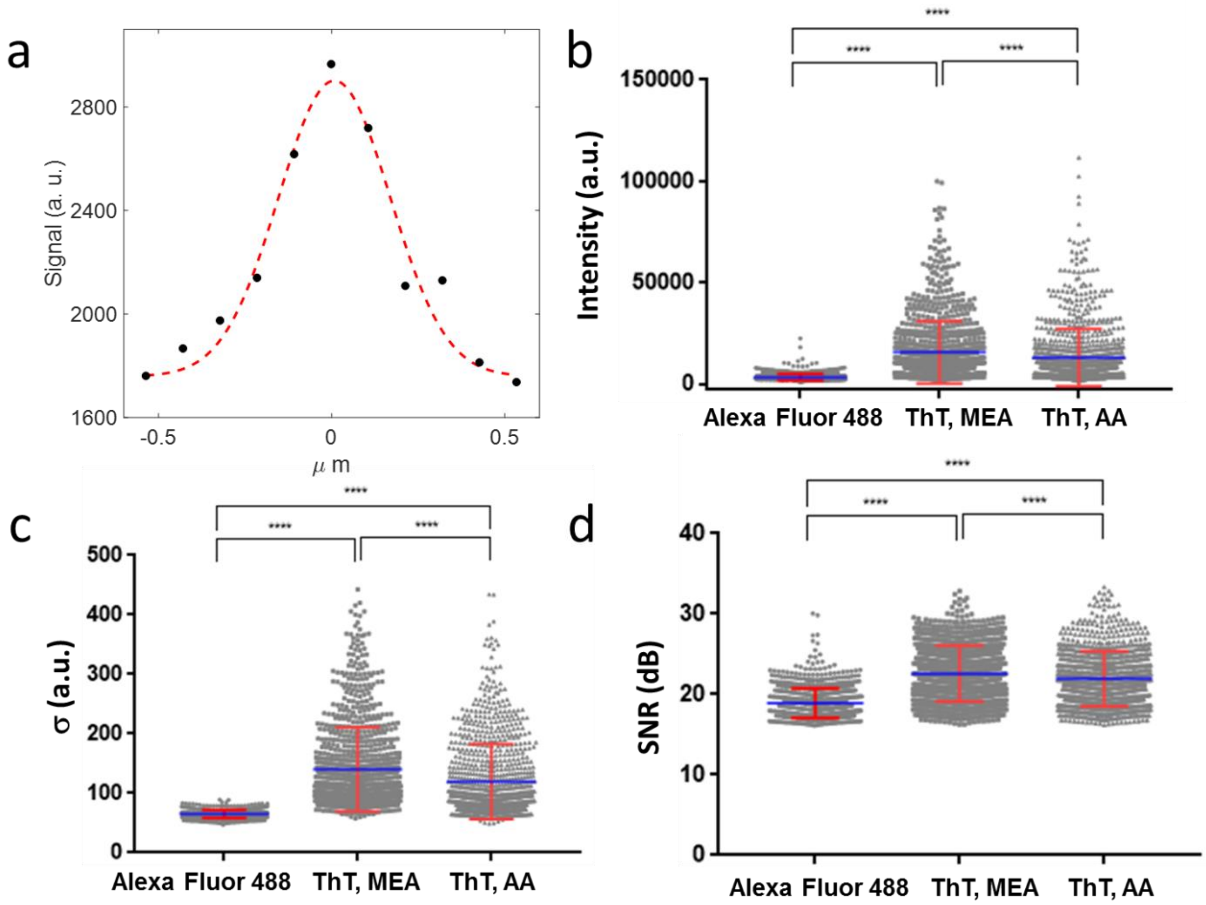


**Figure S3. Localization precision and dSTORM condition quality.** (a) Typical Gaussian fit obtained for a single ThT molecule bound to amyloid (here in MEA buffer). (b-d) Comparison of intensity per molecule (e.g. integrated signal over the measured PSF), standard deviation of the noise (σ) and signal to noise ratio (SNR) for the different samples studied (6 dSTORM movies per sample are analyzed): ThT bound to amyloids in the two photo-switching buffers MEA and AA, and Alexa Fluor 488 deposited on a coverslip in MEA. The total number of molecules retrieved per condition is *n* = 1000. ANOVA tests results of significant differences are represented by (*: $p \leq 0.05$) for statistically significant; (**: $p \leq 0.01$) as highly statistically significant; (***: $p \leq 0.001$) and (****: $p \leq 0.0001$) as extremely statistically significant.

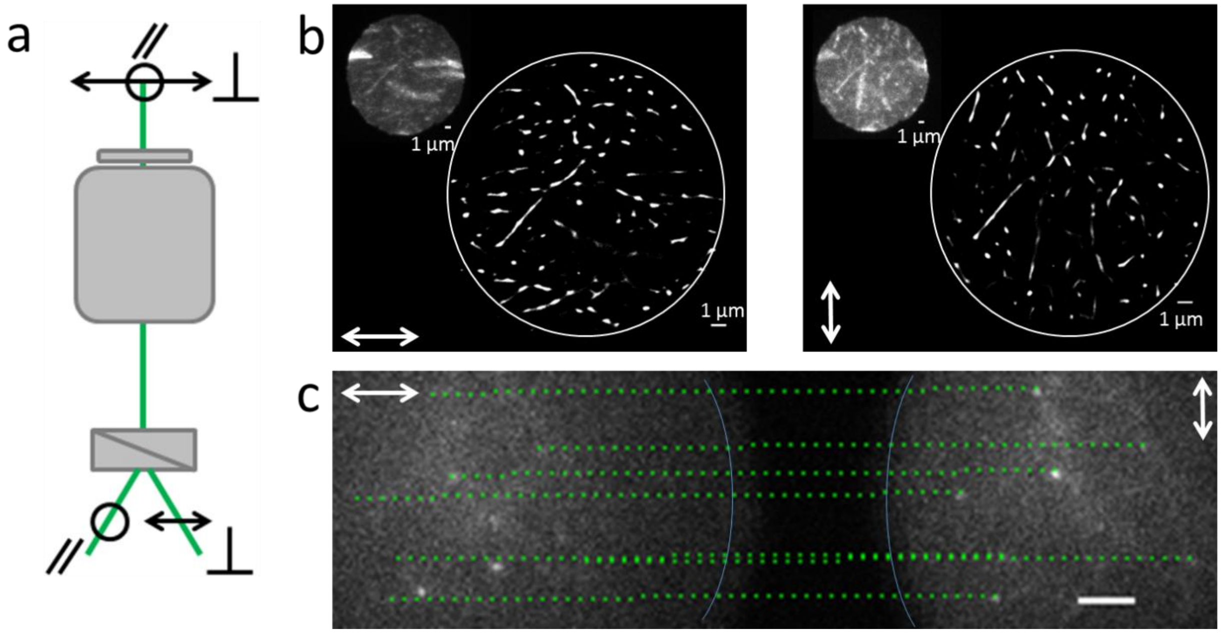


**Figure S4.** P-dSTORM imaging on ThT bound to insulin amyloid fibrils. (a) Principle of the measurement, showing the polarized detection along the horizontal (∥) and vertical (⊥) directions. (b) Retreived wide field image (inset) and reconstructed dSTORM image on both sides of the camera detector. Both types of images evidence that a horizontal polarized detection favors intensities of horizontally oriented fibrils, similarly for vertical polarized detection and fibrils orientations. This indicates a high order of molecules along the fibril direction. (c) Typical P-dSTORM image showing paired single detection events on both sides of the camera, corresponding to ∥ and ⊥ polarized directions. The dashed green lines show the correspondence vector used to pair single molecules (as performed in[2]), in order to determine from each single molecule its polarization factor $P$. The border lines are shown as an eye guide to visualize the two polarized images on the camera chip. Scale bar: 2µm.

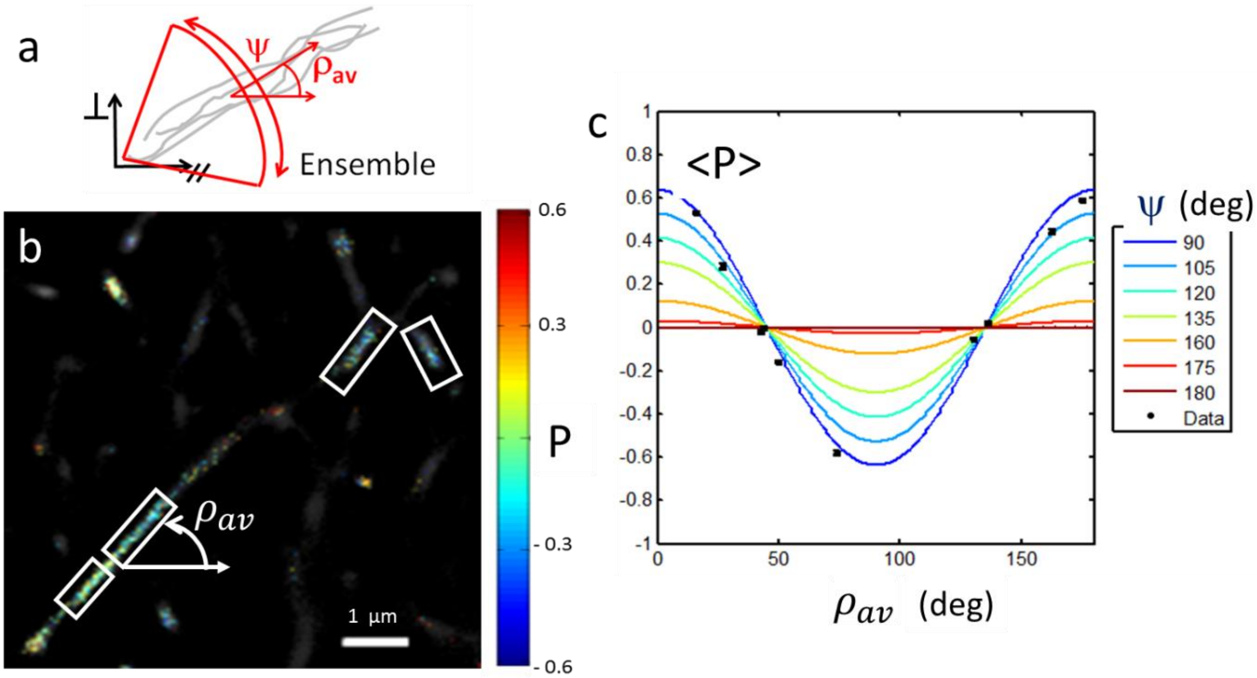


**Figure S5**. Ensemble polarized information gained from P-dSTORM imaging. (a) Principle of the information retrieved from P-dSTORM imaging. An ensemble of single filaments labelled with bound fluorescence molecules is characterized by an averaged fluorophores direction $\rho_{av}$ and a global orientational order reported by the angular extent experienced by fluorophores: $\Psi$. These quantities are 2D parameters representative of a distribution lying in the sample plane. (b) P-dSTORM image depicting $P$ values for single detected ThT molecules, with portions of fibrils identified by their orientation $\rho_{av}$. Only the molecules for which the intensity level is above 20 000 are processed for $P$ calculation. (c) For each portion of fibrils, averaged $P$ values are reported as a function of the fibril orientation $\rho_{av}$. The data are in good agreement with an ensemble molecular order of $\Psi$ ~ 95°, which is close to values previously reported using confocal polarized microscopy[7].

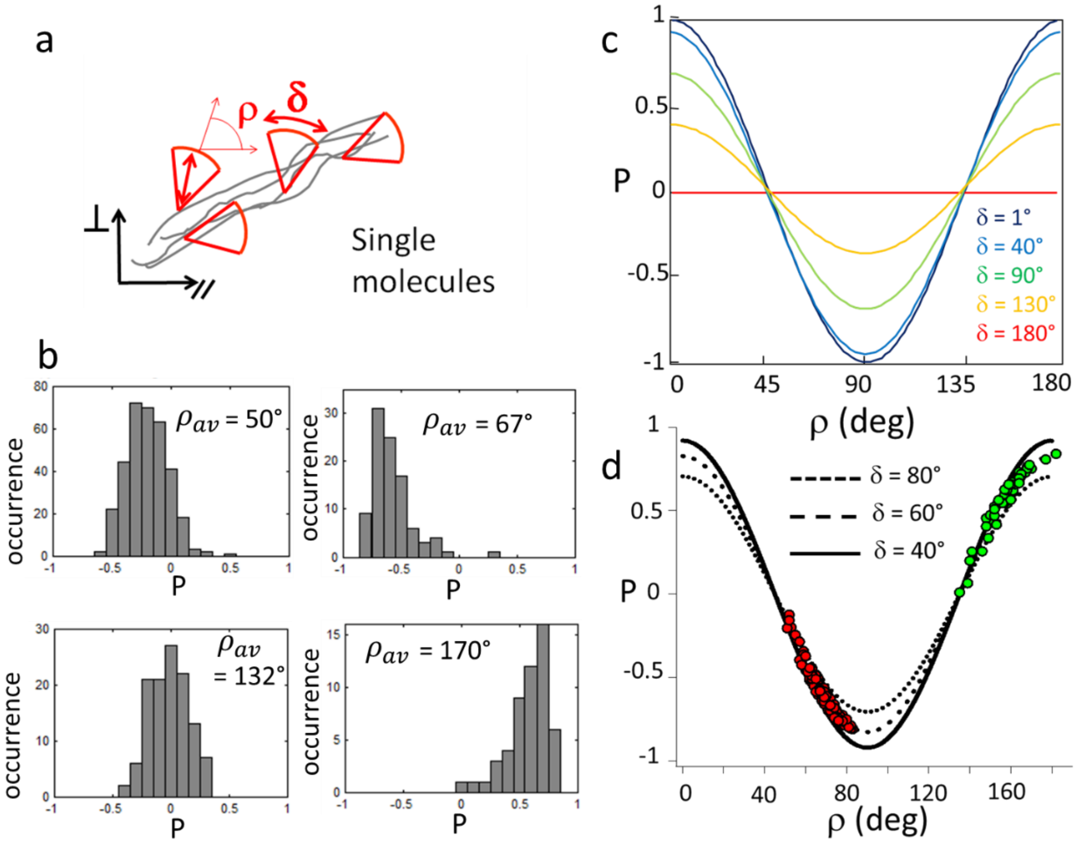


**Figure S6.** Molecular order probed by P-dSTORM single molecule information. (a) Schematic representation of single molecules bound to fibrils, showing for each of them their averaged direction ρ (in the sample plane) and angular extent of fluctuations δ. Only molecules wobbling in a cone lying in the sample plane are represented, as tilted molecules are less efficiently detected for the numerical aperture used in the experiment[2]. (b) Histograms of polarization factor *P* values obtained on portions of fibrils of known orientation $\rho_{av}$, showing a strong dependence of the histogram average as a function of the fibril orientation. (c) Dependence of *P* with respect to the parameters ρ and δ, following the equation $P = \cos 2\rho \,.\, \sin\delta / \delta$ [2]. The extent of *P* values experienced by single molecules (represented by the dashed rectangles), in particular for horizontal and vertical fibrils, permits to deduce a lower limit for the wobbling angle δ. Using in particular the maximum reached value $P_{max}$ and following the equation $P_{max} = \sin\delta / \delta$ leads to $\delta \sim 60°$ [2].(d) Two examples of distribution of measured P values on regions of amyloid fibrils (typically 130 molecules per region). Each dot represent one molecule P values affected by noise to emphasize the error made on the measurement as expected from noise. The noise model is the result of the methodology developed in ref.[2], which follows a square

root Poisson-like model corrected by a factor that depends on the CCD gain used. According to simulations that follow this noise model, a 3° error on $\rho$ is produced for 50 000 signal CCD counts measured. The measured P values lead to an expected $\rho$ which depends on $\delta$. From the maximum P values measured for a given fibril direction, the upper limit for $\delta$ is deduced and hence the $\rho$ values plotted. From all fibrils measured, in particular the ones close to horizontal and vertical directions, the most probable lower value for $\delta$ is 60°.

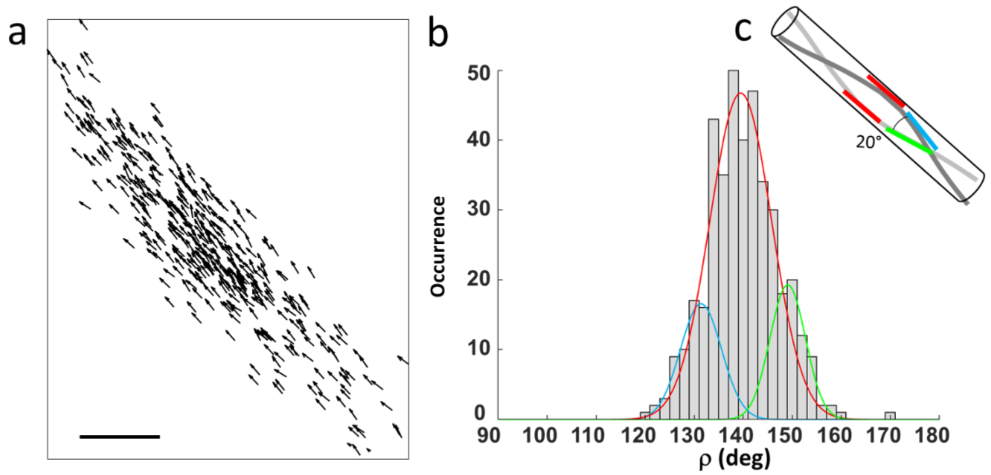


**Figure S7.** P-dSTORM data on a single ThT bound insulin amyloid fibril portion. (a) For each detected single molecule, its mean direction ρ (see Figure S6a) is obtained from the wobbling mean angle value δ (see Figure S6c) using the equation $\rho = \frac{1}{2}\,\mathrm{acos}(P\ \delta / \sin\delta)$ [2]. The arrows are present for visualization. Scale bar: 100 nm. (b) Histogram of the ρ values obtained on the image in (a). A fit using Gaussian functions shows different populations, with maxima located at pointing directions are distant in total by about 20°. The parameters of the Gaussian fits are (in increasing $\rho$ order, giving their center and full width half maximum): 131°(FWHM 10°); 139°(15°);149°(8°)). These directions are schematically represented in a model where ThT follows the direction of individual filaments which twist around each other as in an helical contour, such as predicted in amyloid supra-structures[8,9]. The 20° angle between the found populations could correspond to the most prominent directions visible when the helical structure is projected in the sample plane, the central part of the histogram corresponding to the border of the helix. The found angle is consistent with the pitch obtained from atomic force microscopy or electron microscopy images in reported amyloids of low order structure[8,9]. (c) The measured orientations are schematically represented on an helical structure whose dimension fits with the measured preferential directions, showing a twisted helical geometry with a 20° angular aperture.

**References**


(1) Sergé, A.; Bertaux, N.; Rigneault, H.; Marguet, D. *Nat. Methods* **2008**, *5*, 687–694.

(2) Cruz, C. A. V.; Shaban, H. A.; Kress, A.; Bertaux, N.; Monneret, S.; Mavrakis, M.; Savatier, J.; Brasselet, S. *Proc. Natl. Acad. Sci.* **2016**, *113* (7), E820–E828.

(3) Shroff, H.; Galbraith, C. G.; Galbraith, J. A.; Betzig, E. *Nat. Methods* **2008**, *5*, 417–423.

(4) Dempsey, G. T.; Vaughan, J. C.; Chen, K. H.; Bates, M.; Zhuang, X. *Nature Methods*. 2011, pp 1027–1036.

(5) Song, L.; Hennink, E. J.; Young, I. T.; Tanke, H. J. *Biophys. J.* **1995**, *68*, 2588–2600.

(6) van de Linde, S.; Krstić, I.; Prisner, T.; Doose, S.; Heilemann, M.; Sauer, M. *Photochem. Photobiol. Sci.* **2011**, *10*, 499–506.

(7) Duboisset, J.; Ferrand, P.; He, W.; Wang, X.; Rigneault, H.; Brasselet, S. *J. Phys. Chem. B* **2013**, *117* (3), 784–788.

(8) Jiménez, J. L.; Nettleton, E. J.; Bouchard, M.; Robinson, C. V; Dobson, C. M.; Saibil, H. R. *Proc. Natl. Acad. Sci. U. S. A.* **2002**, *99*, 9196–9201.

(9) Fitzpatrick, A. W. P.; Debelouchina, G. T.; Bayro, M. J.; Clare, D. K.; Caporini, M. A.; Bajaj, V. S.; Jaroniec, C. P.; Wang, L.; Ladizhansky, V.; Müller, S. A.; MacPhee, C. E.; Waudby, C. A.; Mott, H. R.; De Simone, A.; Knowles, T. P. J.; Saibil, H. R.; Vendruscolo, M.; Orlova, E. V.; Griffin, R. G.; Dobson, C. M. *Proc. Natl. Acad. Sci. U. S. A.* **2013**, *110* (14), 5468–5473.
